# Copeptin as a Biomarker in Chronic Kidney Disease—A Systematic Review and Meta-Analysis

**DOI:** 10.3390/biom15060845

**Published:** 2025-06-10

**Authors:** Gabi Gazi, Robert Cristian Cruciat, Daniel-Corneliu Leucuta, Nahlah Al Srouji, Stefan-Lucian Popa, Mohamed Ismaiel, Dinu Iuliu Dumitrascu, Abdulrahman Ismaiel

**Affiliations:** 1 Faculty of Medicine, “Iuliu Hatieganu” University of Medicine and Pharmacy, 400006 Cluj-Napoca, Romania; gabigazi99@gmail.com (G.G.); robert.cruciat10@gmail.com (R.C.C.); 2 Department of Medical Informatics and Biostatistics, “Iuliu Hatieganu” University of Medicine and Pharmacy, 400349 Cluj-Napoca, Romania; 3 2nd Department of Internal Medicine, “Iuliu Hatieganu” University of Medicine and Pharmacy, 400006 Cluj-Napoca, Romania; nahlah.alsrouji@yahoo.com (N.A.S.); popa.stefan@umfcluj.ro (S.-L.P.); abdulrahman.ismaiel@yahoo.com (A.I.); 4 Department of General Surgery, Altnagelvin Hospital, Londonderry BT47 6LS, UK; dr.mohamed.sami.i@gmail.com; 5 Department of Anatomy, “Iuliu Hatieganu” University of Medicine and Pharmacy, 400006 Cluj-Napoca, Romania; d.dumitrascu@yahoo.com

**Keywords:** biomarker, chronic kidney disease, copeptin, glomerular filtration rate

## Abstract

**Background**: Numerous studies have explored the potential of the biomarker copeptin (CPP) in diagnosing and assessing the severity of chronic kidney disease (CKD). Despite these efforts, findings have been inconsistent. Consequently, this study aimed to examine the association between CPP and CKD, specifically evaluating its diagnostic value and correlation with CKD severity as classified by the Kidney Disease Improving Global Outcomes (KDIGO) guidelines. **Methods**: A systematic search of PubMed, EMBASE, and Scopus was conducted using a predefined search string to identify relevant studies. Eligible studies included those involving CKD patients classified by glomerular filtration rate (GFR) according to the Kidney Disease Outcomes Quality Initiative (KDOQI) guidelines or by the estimated GFR (eGFR) calculated using the MDRD formula, provided they met predefined inclusion criteria. Study quality was assessed using the Newcastle–Ottawa Scale (NOS). The primary outcome measured was the mean difference (MD) in serum CPP levels across the various stages of CKD. **Results**: A total of seven studies, comprising 2769 participants, met the inclusion criteria and were incorporated into our systematic review and meta-analysis. Notable differences in CPP levels were identified across various comparisons. Specifically, CPP levels were significantly elevated in CKD patients compared to healthy controls, with a mean difference (MD) of 12.975 (95% CI 6.572, 19.379). Additional significant MDs were observed in comparisons including controls versus CKD stages 1–2/2 (−1.600 [95% CI −3.179, −0.020]), controls versus CKD stage 3 (−9.598 [95% CI −12.959,−6.237]), controls versus CKD stages 4–5 (−28.776 [95% CI −42.925, −14.628]), and CKD stages 1–2 versus stages 4–5 (−30.475 [95% CI −46.790, −14.160]). **Conclusions**: Comparison between the CKD patients and healthy controls revealed significantly elevated CPP levels, suggesting a possible role in renal pathology. Furthermore, the distinct differences in CPP concentrations across various CKD stages highlight its potential as a biomarker for assessing disease severity and progression.

## 1. Introduction

Chronic kidney disease (CKD) represents a major global public health issue, marked by the gradual deterioration of kidney function over time. Its prevalence continues to grow, with millions affected worldwide. CKD imposes a considerable strain on healthcare systems, largely due to its links with heightened morbidity, mortality, and rising medical expenses. Additionally, CKD is frequently associated with complications such as cardiovascular disease, anemia, and bone disorders, all of which contribute to a significant decline in patients’ quality of life [1].

In clinical practice, the timely and accurate diagnosis of CKD is paramount for effective management and intervention strategies. Currently, diagnosis primarily relies on assessing kidney function through the measurements of serum creatinine and estimated glomerular filtration rate (eGFR) [2]. However, these conventional biomarkers may not fully capture the intricacies of renal function and the underlying pathophysiological processes in CKD. Consequently, there is a growing interest in exploring novel biomarkers that could enhance the early detection, risk stratification, and monitoring of CKD progression.

Copeptin (CPP), a stable peptide derived from the precursor of arginine vasopressin (AVP), has gained attention as a potential biomarker in this context. As the C-terminal segment of the AVP precursor, CPP acts as a reliable surrogate marker for AVP secretion [3]. It plays a crucial role in regulating water homeostasis and osmotic balance, primarily through its involvement in the control of renal water reabsorption. Moreover, CPP has been implicated in various pathological conditions, including heart failure, sepsis, and renal dysfunction [4,5]. Its stability in circulation and easy measurement makes it an attractive biomarker for assessing renal function and predicting outcomes in CKD [6].

In the context of CKD, CPP has garnered increasing attention for its potential role as a diagnostic and prognostic marker, as well as a therapeutic target. Studies have suggested associations between elevated CPP levels and adverse outcomes in CKD patients, including progression to end-stage renal disease (ESRD), cardiovascular events, and mortality. However, the existing evidence regarding the relationship between CPP and CKD remains inconclusive, with discrepancies across studies. Therefore, there is a need for a comprehensive synthesis of the available literature to elucidate the role of CPP in CKD and its implications for clinical practice.

This systematic review and meta-analysis aimed to assess the existing evidence on CPP levels in individuals with CKD. Specifically, we sought to examine the association of CPP levels in CKD patients compared to healthy controls, as well as among different CKD stages. Unlike previous reviews, our study uniquely included a stratified analysis based on the KDIGO classification, providing a stage-specific evaluation of CPP levels. This enhanced the granularity and potential clinical applicability of the findings. By integrating findings from the published literature, our goal was to evaluate the potential of CPP as a biomarker for monitoring CKD progression, thereby supporting clinical decision-making and shaping future research directions.

## 2. Materials and Methods

This systematic review and meta-analysis was conducted and reported in accordance with the Preferred Reporting Items for Systematic Reviews and Meta-Analyses (PRISMA) 2020 guidelines [7]. It has not been registered.

### 2.1. Data Sources and Strategy

A computerized search was carried out using PubMed, Embase, and Scopus databases to identify observational studies evaluating CPP levels in patients with CKD. The specific search strategy is detailed in Supplementary Material S1. Additionally, a manual search was performed by screening the reference lists of the included articles to capture any potentially missed studies. The literature search spanned from database inception to 8 December 2023, and was independently conducted by two reviewers (G.G. and R.C.C.). Any disagreements were resolved through discussion to reach consensus. No filters or restrictions were applied with regards to the publication date, country, or language. Titles and abstracts were initially screened for relevance, followed by a full-text review based on predefined inclusion and exclusion criteria. Data extraction was independently performed by two reviewers (G.G. and R.C.C.) and cross-checked by a third investigator (A.I.); discrepancies were resolved by consulting the original sources. Extracted information included author names, publication year, study location, study design, characteristics of the study population, total sample size, the percentage of CKD cases, mean age, gender distribution, body mass index (BMI), CKD diagnostic method, CPP levels (expressed as mean ± standard deviation or median with interquartile range), and primary outcomes. These data were compiled and are presented in the manuscript.

### 2.2. Eligibility Criteria

Original studies were included in this systematic review and meta-analysis based on the following criteria: (1) observational designs—specifically cohort, cross-sectional, or case-control studies—investigating the circulating levels of CPP in individuals with CKD; (2) the diagnosis of CKD as defined by the respective study protocols; (3) research conducted in human subjects, with no exclusions based on sex, race, or ethnicity; and (4) articles published in English, Swedish, German, French, or Romanian.

Studies were excluded based on the following criteria: (1) a lack of reporting on CPP levels stratified by CKD status, control groups, or KDIGO classification; and (2) non-original research formats, including editorials, letters, brief surveys, commentaries, case reports, conference abstracts, review articles, pediatric studies, clinical practice guidelines, and abstracts without an accompanying full-text article.

### 2.3. Risk of Bias Assessment in Individual Studies

Two investigators (G.G. and R.C.C.) independently assessed the risk of bias and internal validity of the included studies using the Newcastle–Ottawa Scale (NOS) [8]. Any disagreements regarding quality assessments were resolved through discussion. Each study was rated based on the number of stars awarded across three domains: selection, comparability, and outcome (or exposure for case-control studies). Scores ranged from 0 to 10 stars, and the total star count was used to facilitate the quantitative comparison of study quality. Studies receiving a score of 7 or more stars were classified as high quality. Importantly, the quality assessment did not influence the inclusion or exclusion of studies in the analysis.

### 2.4. Summary Measures and Synthesis of Results

Data analyses for the systematic review and meta-analysis were conducted using R software with the Metafor package (OpenMeta Analyst) [9]. The primary outcome measure was the mean difference (MD) in circulating CPP levels among individuals with CKD. Between-study heterogeneity was assessed using the chi-squared (χ^2^)-based Q-test and the I^2^ statistic. Following the Cochrane Handbook guidelines for interpreting heterogeneity, I^2^ values were classified as follows: 0–40% (likely unimportant), 30–60% (moderate), 50–90% (substantial), and 75–100% (considerable heterogeneity) [10]. For studies that reported medians and interquartile ranges (IQRs), the mean and standard deviation (SD) were estimated using established statistical methods. In studies with multiple CPP or control subgroups, group means and SDs were aggregated to generate values representative of the entire cohort, in accordance with Cochrane recommendations. Subgroup analyses were performed based on CKD severity, as defined by the KDIGO classification, using data extracted from the included studies. All meta-analyses were performed using restricted maximum likelihood random-effects models. For each study, results were presented as MDs with corresponding 95% confidence intervals (CIs), standard error values, and *p*-values. Statistical significance was defined as a *p*-value < 0.05. Analyses were conducted only when at least two studies reported the same outcome with an available mean and SD, or median and IQR.

## 3. Results

### 3.1. General Results

**Figure 1:**
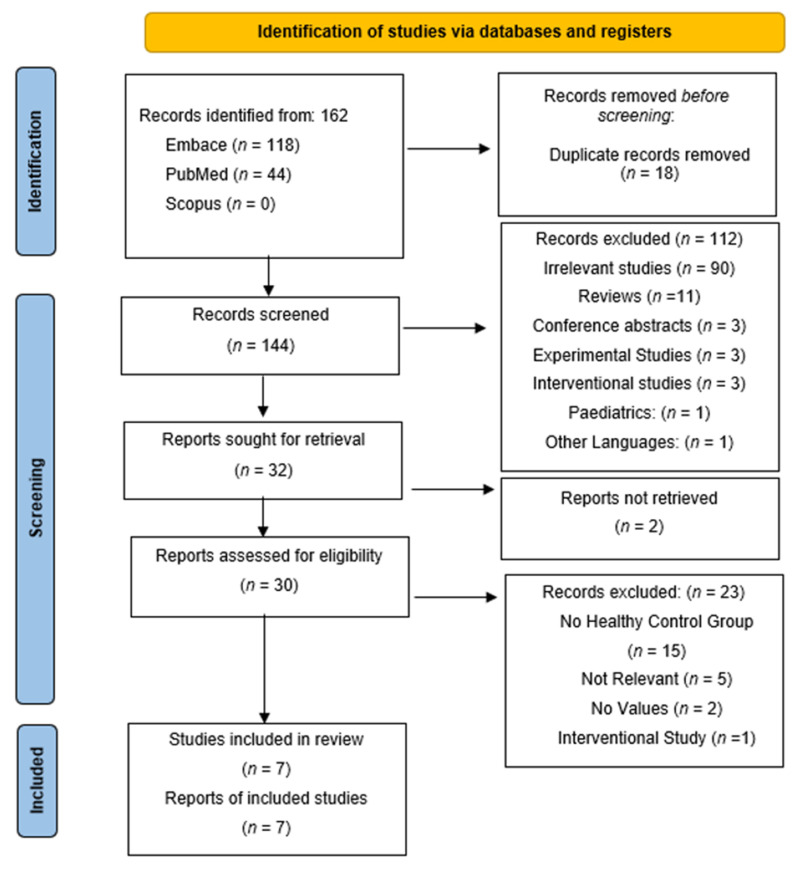
RISMA flow diagram illustrating the identification, screening, eligibility, and inclusion phases of this systematic review and meta-analysis.

A total of 165 articles were initially identified through database searches—118 from PubMed, 44 from EMBASE, and none from Scopus, as depicted in Figure 1. Eighteen duplicate entries were removed, leaving one hundred and forty-four articles for title and abstract screening to assess eligibility based on predefined inclusion and exclusion criteria. Following this initial screening, one hundred and twelve articles were excluded for the following reasons: ninety were review articles, three were conference abstracts, three were interventional studies, three were experimental studies, one involved a pediatric population, one was an editorial, and eleven were unrelated to the research topic. Additionally, two articles could not be retrieved. Full-text evaluation was then performed on the remaining 30 articles. Of these, twenty-three were excluded for the following reasons: (1) Fifteen did not include healthy control groups [11,12,13,14,15,16,17,18,19,20,21,22,23,24,25], (2) five were irrelevant to the study aim [16,26,27,28,29], (3) two did not report CPP measurements [30,31], (4) two not retrieved [32,33], (5) and one was an interventional study [34]. Ultimately, seven studies met all criteria and were included in the qualitative synthesis, all of which were also incorporated into the quantitative synthesis [35,36,37,38,39,40,41].

### 3.2. Study Characteristics

A detailed summary of the key characteristics of the included studies is provided in Supplementary Table S1. This systematic review and meta-analysis encompassed a total of 2769 participants. Excluding one study that did not report sex distribution, the majority of participants were female, with 1444 females (53.7%) and 1249 males (46.3%). Among the total study population, 1453 individuals (51.8%) were diagnosed with CKD. Geographically, three studies were conducted in Asia (Japan *n* = 1, China *n* = 1, Iraq *n* = 1), three in Europe (Germany *n* = 1, Poland *n* = 1, Sweden *n* = 1), and one in North America (Mexico *n* = 1).

### 3.3. Definition of CKD

CKD was assessed using eGFR or GFR for diagnosing in all studies (*n* = 7).

### 3.4. CPP Levels in CKD

#### 3.4.1. CPP Levels in CKD Patients vs. Controls

**Figure 2:**
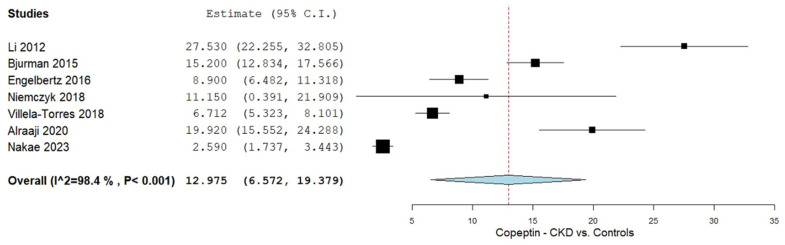
CPP levels in CKD patients vs. controls [35,36,37,38,39,40,41].

CPP levels were analyzed across seven studies that compared values between the CKD patients and control groups [35,36,37,38,39,40,41]. A summary of the resulting meta-analysis is presented in Figure 2. The combined analysis evaluating CPP levels in adult CKD patients versus controls yielded an overall mean difference (MD) of 12.975 (95% CI 6.572, 19.379). Substantial heterogeneity was observed, with an I^2^ of 98.4% and a *p*-value of <0.001.

#### 3.4.2. Controls vs. CKD Stages (1–2/2, 3, 4, 5, and 4–5)

**Figure 3:**
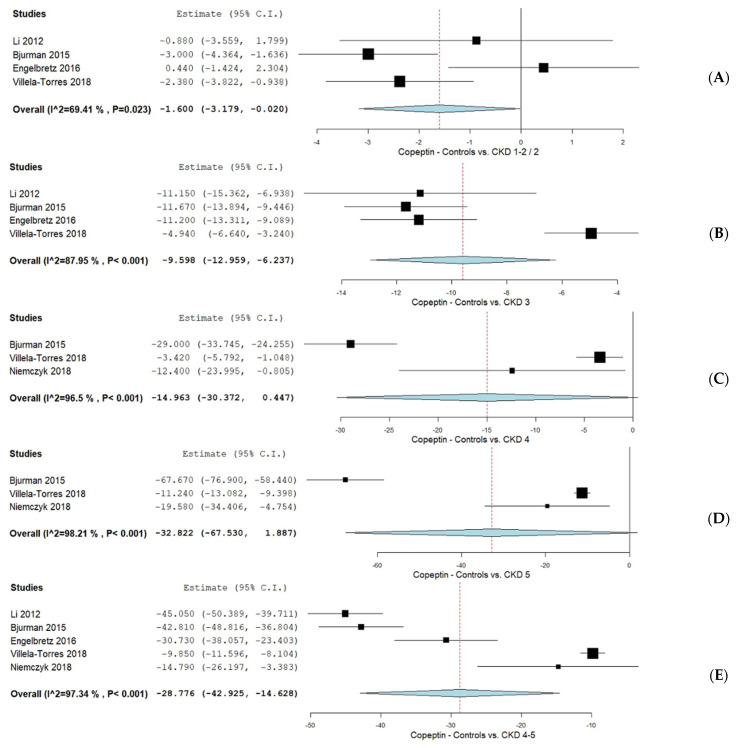
CPP levels in CKD patients according to controls vs. KDIGO CKD classification. (**A**) CPP levels in controls vs. KDIGO CKD stages 1–2/2; (**B**) CPP levels in controls vs. KDIGO CKD stages 3; (**C**) CPP levels in controls vs. KDIGO CKD stage 4; (**D**) CPP levels in controls vs. KDIGO CKD stage 5; (**E**) CPP levels in controls vs. KDIGO CKD stage 4–5 [36,38,39,40,41].

Additionally, subgroup analyses were performed in adult populations based on CPP levels across the different KDIGO CKD stages, compared to control groups, as illustrated in Figure 3.

CPP levels in control subjects vs. those with KDIGO CKD stages 1–2/2 were analyzed in four studies [36,37,38,41], yielding a mean difference (MD) of −1.600 (95% CI −3.179, −0.020), with substantial heterogeneity (I^2^ = 69.41%, *p* = 0.023). For KDIGO CKD stage 3, CPP levels were also examined in four studies [36,37,38,41], showing an MD of −9.598 (95% CI −12.959, −6.237) and considerable heterogeneity (I^2^ = 87.95%, *p* < 0.001). Three studies [36,40,41] evaluated CPP levels in controls versus patients with KDIGO CKD stage 4, reporting an MD of −14.963 (95% CI −30.372, 0.447), accompanied by considerable heterogeneity (I^2^ = 96.5%, *p* < 0.001). For stage 5, three studies [36,40,41] reported an MD of −32.963 (95% CI −67.530, 1.887), again with considerable heterogeneity (I^2^ = 98.21%, *p* < 0.001). Additionally, a pooled analysis from five studies [36,37,38,40,41] comparing controls to patients with KDIGO CKD stages 4–5 indicated an MD of −28.776 (95% CI −42.925, 14.628) with considerable heterogeneity (I^2^ = 97.34%, *p* < 0.001).

#### 3.4.3. CPP Levels in CKD Patients According to the KDIGO CKD Classification

**Figure 4:**
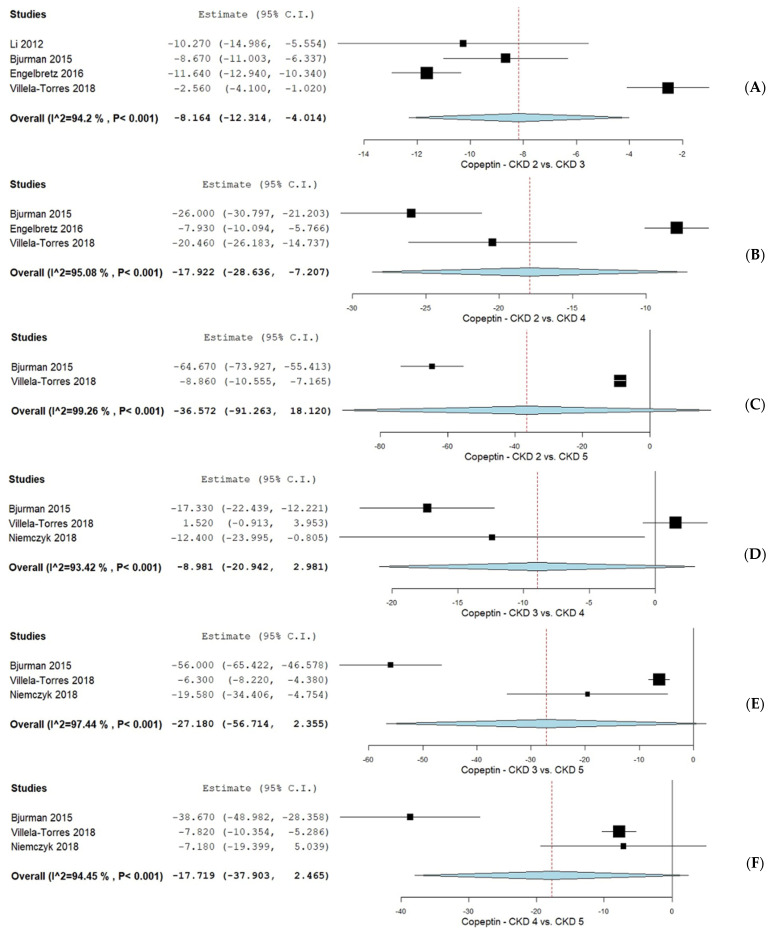
CPP levels in CKD patients according to KDIGO CKD classifications. (**A**) CPP levels KDIGO CKD stage 2 vs. KDIGO CKD stage 3; (**B**) CPP levels in KDIGO CKD stage 2 vs. KDIGO CKD stage 4; (**C**) CPP levels in KDIGO CKD stage 2 vs. KDIGO CKD stage 5; (**D**) CPP levels in KDIGO CKD stage 3 vs. KDIGO CKD stage 4; (**E**) CPP levels in KDIGO CKD stage 3 vs. KDIGO CKD stage 5; (**F**) CPP in KDIGO CKD stage 4 vs. KDIGO CKD stage 5 [36,38,39,40,41].

As shown in Figure 4, a subgroup analysis was performed based on CPP levels across different KDIGO CKD stages. CPP levels in CKD stage 2 versus stage 3 were assessed in four studies [36,37,38,41], revealing a mean difference (MD) of −8.164 (95% CI −12.314, 4.014) with considerable heterogeneity (I^2^ = 94.2%, *p* < 0.001). The comparison between CKD stages 2 and 4, evaluated in three studies [36,37,41], showed an MD of −17.922 (95% CI −28.636, −7.207) and considerable heterogeneity (I^2^ = 95.08%, *p* < 0.001). Two studies [36,41] assessed CPP levels between CKD stages 2 and 5, reporting an MD of −36.572 (95% CI −91.263, 18.120) with considerable heterogeneity (I^2^ = 99.26%, *p* < 0.001). For CKD stage 3 versus stage 4, three studies [36,40,41] reported an MD of −8.981 (95% CI −20.942, 2.981) with considerable heterogeneity (I^2^ = 93.42%, *p* < 0.001). The comparison between CKD stages 3 and 5, also in three studies [36,40,41], showed an MD of −27.180 (95% CI −56.714, 2.355), with considerable heterogeneity (I^2^ = 97.44%, *p* < 0.001). Finally, CPP levels between CKD stages 4 and 5 were evaluated in three studies [36,40,41], yielding an MD of −17.719 (95% CI −37.903, 2.465) and considerable heterogeneity (I^2^ = 94.45%, *p* < 0.001).

#### 3.4.4. CKD Patients According to KDIGO CKD Classification

**Figure 5:**
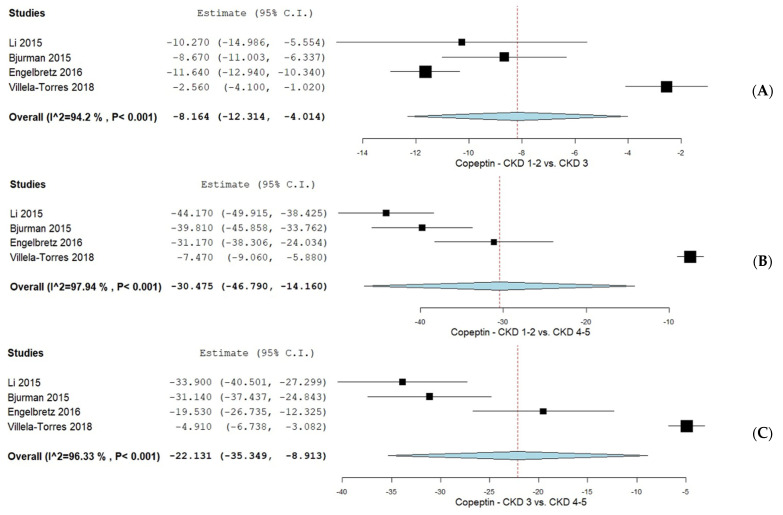
(**A**) CPP levels in CKD stages 1–2 vs. CKD stage 3; (**B**) CPP levels in CKD stages 1–2 vs. CKD stages 4–5; (**C**) CPP levels in CKD stage 3 vs. CKD stages 4–5 [36,38,39,40,41].

As illustrated in Figure 5, an additional subgroup analysis was performed based on CPP levels across KDIGO CKD classifications. CPP levels in patients with CKD stages 1–2 compared to stage 3 were evaluated in four studies [36,37,38,41], yielding a mean difference (MD) of −8.164 (95% CI −12.314, −4.014) with considerable heterogeneity (I^2^ = 94.2%, *p* < 0.001). The comparison between CKD stages 1–2 and stages 4–5, assessed in four studies [36,37,38,41], showed an MD of −30.475 (95% CI −46.790, −14.160) and considerable heterogeneity (I^2^ = 97.94%, *p* < 0.001). CPP levels between CKD stage 3 and stages 4–5 were also analyzed in four studies [36,37,38,41], with an MD of −22.131 (95% CI −35.349, −8.913) and considerable heterogeneity (I^2^ = 96.33%, *p* < 0.001).

### 3.5. Bias Evaluation

The methodological quality of the studies included in this systematic review and meta-analysis was assessed using the NOS quality assessment tool, as presented in Supplementary Table S2. Seven articles in total were evaluated using the NOS criteria specifically designed for cross-sectional studies [8,35,36,37,38,39,40,41].

One study received an overall NOS quality rating of 8/10 [37], two studies were rated 7/10 [38,39], one study was rated 6/10 [36], and three studies received a rating of 5/10 [35,40,41]. All studies clearly stated their research objective or question. In four of the studies, the population sample was either truly or somewhat representative of the average target population, and the sample size was both adequate and appropriately justified [36,37,38,39]. Each study utilized a validated measurement tool. All seven studies controlled for the most important confounding factor and at least one additional factor. Furthermore, outcome assessment was conducted via record linkage in all included studies, and each employed an appropriate, clearly described statistical test with adequately reported results.

## 4. Discussion

In recent years, various scores and biomarkers have been investigated in the context of CKD, with the aim of enhancing diagnostic accuracy and identifying novel biomarkers. In this systematic review and meta-analysis, we examined CPP levels in CKD patients, stratified by KDIGO CKD classification. A total of seven studies, encompassing 2769 participants, were included in both the quantitative and qualitative analyses. Our findings indicated that CPP levels were significantly elevated in adult CKD patients compared to the control subjects.

The diagnosis and classification of CKD are pivotal for effective management and prognostication. Conventionally, estimating the glomerular filtration rate eGFR or directly measuring the GFR serve as key diagnostic criteria. In recent research, the focus has intensified on refining these approaches through the exploration of novel scores and biomarkers. The variability in diagnostic approaches is essential for understanding the clinical relevance and applicability of the biomarkers under investigation across different scenarios. According to current guidelines, eGFR calculation or direct GFR measurement is recommended when there is clinical suspicion of CKD. Diagnosing CKD entails a structured approach. Initially, assessing risk factors and symptoms can guide screening. Following this, calculating eGFR through equations like the Modification of Diet in Renal Disease (MDRD) or Chronic Kidney Disease Epidemiology Collaboration (CKD-EPI) equation is crucial. Additionally, evaluating urine albumin-to-creatinine ratio aids in diagnosis. Subsequently, kidney imaging and biopsy may be warranted for further characterization. Integrating these steps facilitates the timely and accurate diagnosis of CKD, enabling prompt intervention and improved patient outcomes [2].

One disease that is frequently combined with a decline in renal function is type 2 diabetes mellitus (T2DM). This represents significant health challenges globally, especially with the prevalence steadily rising. In this landscape, understanding the pivotal role of CPP emerges as a crucial aspect of effective management and prognosis for individuals grappling with these conditions. CPP, a stable byproduct of the vasopressin precursor, has garnered increasing attention due to its multifaceted implications in various physiological processes, especially in the context of CKD and T2DM [42]. Its utility extends beyond a mere biomarker. It serves as a sentinel for renal function, cardiovascular health, and metabolic regulation [25]. In CKD patients, elevated CPP levels often mirror the severity of renal impairment, offering clinicians invaluable insights into disease progression and prognosis. In two articles published by Velho G et al., the authors mentioned that plasma CPP levels were additionally linked to the risk of severe kidney complications, such as a twofold increase in plasma creatinine concentration and/or the onset of end-stage renal disease (ESRD), among individuals with either type 1 or type 2 diabetes mellitus [24,25]. This highlights its importance, especially its association with fluid balance regulation, which underscores its utility in guiding fluid management strategies, thereby mitigating the risk of volume overload and its detrimental consequences [42].

In our meta-analysis, we observed a significant elevation in CPP levels among patients with CKD stages 1, 2, and 3 when compared to healthy controls. The underlying mechanisms and potential effects of increased CPP in the context of CKD have been explored and discussed in multiple published studies. The exact mechanism is not fully understood yet. However, two mechanisms could possibly explain this phenomenon, as described in a study conducted by Afsar et al. [6]. The authors reported that CPP is cleared by kidney excretion and the levels of CPP tend to increase as kidney function decreases. Another mechanism explained by the authors is that in patients with lower kidney function, more CPP is released because of the arginine vasopressin (AVP) system being activated due to an impaired urine concentrating capacity to maintain water homeostasis [6]. In another study published by Iglesias et al., CPP, a glycopeptide consisting of 39 amino acids, is released in equal amounts with AVP from the preprohormone AVP within the posterior pituitary [43]. While AVP poses challenges due to its instability and technical difficulties in measurement, CPP stands out as a stable and easily measurable molecule. Consequently, circulating CPP serves as a substitute for AVP in various pathological conditions, including renal diseases. Recent studies have revealed an association between CPP and an elevated risk of CKD development in the general population [6]. To emphasize this more, in an article published by Pikkemaat et al., the authors stated that there is an association between CPP levels and declining GFR in newly diagnosed diabetic patients [11]. This shows that our hypothesis regarding the etiology of CKD shows that CPP has a major role in the pathophysiology of the disease progression.

The observation that CPP levels showed a significant increase between controls vs. CKD stages 1, 2, and 3 separately but not in stages 4 and 5 separately could be attributed to several factors related to the pathophysiology of CKD mentioned above regarding the AVP system. In an article by Mejer et al., the authors suggested that the partial renal clearance of CPP is unlikely to account for the elevated CPP levels observed in individuals with reduced GFR. They argued that an increase in CPP levels would decrease plasma osmolarity, which in turn would result in a subsequent reduction in CPP concentration [44]. Another explanation is the non-linear relationship. It is also plausible that the relationship between CPP and CKD severity follows a non-linear pattern. This means that while there is a clear association and increase in CPP levels in early to moderate CKD stages (CKD 1, 2, and 3), this relationship may plateau or change direction in advanced CKD 4 and 5. This could be due to compensatory mechanisms or other physiological changes occurring in the later stages of CKD. Moreover, the sample size of the included studies and CPP sampling variability are other considerations that could be related to CPP levels within each CKD stage group. If the number of patients in stages 4 and 5 was relatively small or if there was significant heterogeneity within these groups (e.g., due to differences in etiology or comorbid conditions), this could have affected the statistical power to detect significant differences [45]. Combining CKD stages 4 and 5 into a single group and comparing them to healthy controls resulted in a significant increase in CPP levels, which could be attributed to an enhanced statistical power to detect differences, particularly if individual analyses of these stages separately did not reach significance due to smaller sample sizes or variability. By pooling these advanced stages, the collective impact of severe renal dysfunction on CPP levels became more apparent. Although our results showed increasing CPP levels with advancing CKD stages, wide confidence intervals in advanced stages suggested variability that warrants cautious interpretation. Further studies are needed to establish definitive stage-specific thresholds.

Moreover, another statistical comparison was conducted by combining and comparing CPP values in CKD patients between combined CKD KDIGO stages. Comparing stages CKD 1–2/2, CKD 3, and CKD 4–5 separately against healthy controls, we observed a marked increase in CPP values. Assessing deeper, intra-CKD analysis, a type of analysis, was performed on CPP levels in CKD patients and revealed intriguing patterns when comparing various CKD stages. Initially, when comparing healthy controls with different CKD stages (CKD 2, CKD 3, CKD 4–5), we observed a significant increase with a consistent increasing pattern in CPP levels as kidney function declined, which went hand in hand with the previous literature linking CPP accumulation to impaired renal clearance. However, a more nuanced analysis within the CKD patient group unveiled additional insights. When comparing CPP levels in between CKD stages (CKD 2 vs. CKD 5, CKD 3 vs. CKD 4, CKD 3 vs. CKD 5, CKD 4 vs. CKD 5), the differences were statistically insignificant, indicating the non-progressive elevation of CPP with worsening renal impairment, while on the other hand, the remaining intra-CKD KDIGO class comparisons that were conducted (CKD 2 vs. CKD 3, CKD 2 vs. CKD 4) showed a statistical significance. Interestingly, when we combined CKD stages into broader categories (CKD 1–2 vs. CKD 3, CKD 1–2 vs. CKD 3–4, CKD 3 vs. CKD 4–5), we observed a significant increase in CPP levels between these composite groups.

These findings highlight the complexity of CPP dynamics in CKD progression. The significant differences observed between adjacent CKD stages underscored the gradual and incremental rise in CPP levels as renal function deteriorates. Notably, the pronounced elevation in CPP when comparing broader CKD stage categories suggested a more substantial shift in CPP secretion during the later stages of CKD. Our study findings, particularly the significant increase in CPP levels observed when combining CKD stages into broader categories, can be attributed to several factors, including the larger sample size and increased statistical power associated with combining groups. By aggregating CKD stages into composite categories, such as CKD 1–2 and CKD 4–5, we effectively increased the sample size within each comparison group. This larger sample size provided greater statistical power and sensitivity, enhancing our ability to detect subtle differences in CPP levels between these broader CKD stage classifications. The inclusion of more study participants across multiple CKD stages strengthened the validity and generalizability of our findings, enabling more robust statistical analyses. It is important to note that not all studies could be included in every comparison due to variations in study design and reporting. Some studies provided pre-defined combined CKD stage groups (e.g., CKD 1–2 combined) [37,38], limiting our ability to conduct direct pairwise comparisons across all individual CKD stages. However, the integration of studies with pre-combined groups into our analysis strategy allowed for a comprehensive assessment of CPP dynamics across a spectrum of CKD severities.

While CPP shows promise as a biomarker in CKD, its implementation in clinical practice must also consider factors such as cost-effectiveness and accessibility. CPP assay availability and pricing vary across healthcare systems and regions, and further health-economic evaluations are needed to determine its practical utility in routine care.

Although our study is comprehensive, it is not without limitations that should be acknowledged. A primary concern was the heterogeneity introduced by the varied characteristics of participants and the diverse etiologies of CKD across the included studies. This methodological and demographic variability may have contributed to inconsistency in our analysis, potentially affecting the overall coherence and generalizability of the results. Due to the limited number of relevant studies available, conducting subgroup analyses based on CKD etiology was not feasible. Additionally, inconsistency in the diagnostic criteria for CKD among the included studies posed a challenge. The use of differing methods, such as estimated GFR versus direct GFR measurement, complicated standardization and may have impacted the accuracy of CKD classification. Another limitation involved the measurement of CPP levels, where methodological differences between studies could result in heterogeneity. Variations in sample handling, assay methods, and calibration standards may have affected the comparability of CPP values, thereby influencing the reliability of our findings. Furthermore, differences in how the studies adjusted for covariates may have also impacted the precision and validity of our estimates regarding the relationship between CPP levels and CKD outcomes. Variations in assay methodology, including differences in calibration, detection limits, and sample handling may have influenced CPP measurements across studies. Standardizing assay protocols and adopting universally accepted measurement kits will be essential for translating CPP testing into routine clinical use. Given these limitations, our findings should be interpreted with caution. While this study contributed meaningful insights into the association between CPP levels and CKD outcomes, additional research is needed to strengthen the reliability and clinical applicability of these conclusions.

Our study also possessed several key strengths that reinforced the validity and relevance of our findings. First, the application of a rigorous methodological framework promoted transparency and thoroughness, thereby increasing the dependability of our results. Furthermore, the inclusion of studies from a wide range of geographic locations and diverse populations broadened the scope and applicability of our conclusions. This inclusive approach enabled a more comprehensive understanding of the relationship between CPP levels and CKD across varying settings, thereby enhancing the generalizability of our findings. By integrating data from multiple sources and employing standardized analytical methods, we improved both the accuracy and consistency of our estimates, yielding valuable insights into the CPP–CKD association. Our analysis was further strengthened by a detailed examination of the potential sources of bias and heterogeneity, including publication bias and methodological differences. Addressing these factors contributed to the credibility and trustworthiness of our conclusions, supporting a well-rounded and objective interpretation of the data. Taken together, our study provided a meaningful contribution to the current body of knowledge on CPP and CKD, offering insights that are relevant to both research and clinical practice. The strengths discussed here underscored the robustness of our approach and the significance of our findings, highlighting the importance of our work in advancing understanding and guiding future investigations in this area.

## 5. Conclusions

In CKD patients, CPP levels were significantly higher compared to those in the healthy control group. This elevation was also found to be significant across the different CKD KDIGO stages. Among the CKD groups, patients classified under KDIGO stages 4–5 exhibited the highest CPP concentrations. Given the consistent elevation of CPP levels across various CKD stages, these findings support the potential utility of CPP as a stage-sensitive biomarker. Future longitudinal studies are warranted to validate its clinical applicability in tracking CKD progression.

## Data Availability

The data analyzed in this study were extracted from the original articles referenced, as detailed in Supplementary Table S1.

## Supplementary Materials

The following supporting information can be downloaded at: https://www.mdpi.com/article/10.3390/biom15060845/s1, File S1: Search Strategy; Table S1: Studies assessing CPP levels in CKD patients; Table S2: The Newcastle-Ottawa Scale (NOS) for assessing the quality of cross-sectional studies.
