# Supplementary material for: Copeptin as a Biomarker in Chronic Kidney Disease—A Systematic Review and Meta-Analysis
Source: Biomolecules. 2025 Jun 10;15(6):845. doi: 10.3390/biom15060845 (PMC12191427; doi:10.3390/biom15060845)
Supplement: Supplementary file 1 [file biomolecules-15-00845-s001.zip › Supplementary Material File 1.pdf]

Supplementary Material 1

Search Strategy

08.12.2023

PubMed: 44 articles

(("copeptins" [Supplementary Concept])) AND (("Renal Insufficiency, Chronic"[Mesh]) OR ("Renal Insufficiency, Chronic"[All Fields]) OR ("Kidney Failure, Chronic"[Mesh]) OR ("Kidney Failure, Chronic"[All Fields]))

EMBASE: 118 articles

('copeptins'/exp) AND ('Renal Insufficiency, Chronic'/exp OR 'Renal Insufficiency, Chronic'/exp OR 'Kidney Failure, Chronic'/exp OR 'Kidney Failure, Chronic')

Scopus: 0 articles

(("copeptins" [Supplementary Concept])) AND (("Renal Insufficiency, Chronic"[Mesh]) OR ("Renal Insufficiency, Chronic"[All Fields]) OR ("Kidney Failure, Chronic"[Mesh]) OR ("Kidney Failure, Chronic"[All Fields]))
