# Supplementary material for: Copeptin as a Biomarker in Chronic Kidney Disease—A Systematic Review and Meta-Analysis
Source: Biomolecules. 2025 Jun 10;15(6):845. doi: 10.3390/biom15060845 (PMC12191427; doi:10.3390/biom15060845)
Supplement: Supplementary file 1 [file biomolecules-15-00845-s001.zip › Supplementary Table S1 CPP.pdf]

Supplementary Table 1. Studies assessing CPP levels in CKD patients

First Author / Year / Country	Study Design	Study Characteristics	Main Findings
**LI et al. / 2012 / China** [38]	Cohort Observational Study	• **Total Subjects:** 106<br>• **Population:** Chinese patients with CKD<br>• **CKD:** 86 (81%)<br>• **Mean age (years):** CKD: 45.87±3.39; Controls: 45.67±13.51<br>• **Sex (males):** 60 (56.6%)<br>• **BMI:** CKD: 24.27±2.27; Controls: 24.24±2.64<br>• **CPP Measurement Method**: ELISA<br>• **CPP (pmol/L)- Mean ± SD:** Controls (*n*=20): 9.21±2.64; CKD KDIGO 1-2 (*n*=18): 10.09±5.23; CKD KDIGO 3 (*n*=21): 20.36±9.47; CKD KDIGO 4-5 (*n*=47) 54.26±18.23<br>• **CPP – AUC: -**<br>• **Measurement:** Plasma	Plasma CPP levels were notably elevated in patients with CKD compared to healthy controls. The highest concentrations of plasma CPP were observed in patients classified as having CKD KDIGO Stage 4-5.
**Bjurman et al. / 2015 / Sweden** [36]	Cohort	• **Total Subjects:** 460<br>• **Population:** Swedish patients with decreased renal function<br>• **CKD:** 378 (82%)<br>• **Mean age (years):** CKD: 56.73±15.32; Controls: 46.33±17.13<br>• **Sex (males):** 262 (57%)<br>• **BMI:** -<br>• **CPP Measurement Method**: ELISA<br>• **CPP (pmol/L)- Mean ± SD:** Controls (*n*=80): 6.33±3.77; CKD Stage 1-2 (*n*=148):9.33 ±6.74; CKD Stage 3 (*n*=146):18.00±12.73; CKD Stage 4 (n=54): 35.33±17.52; CKD Stage 5 (*n*=30): 74.00±25.69<br>• **CPP – AUC: -**<br>• **Measurement:** Plasma	Patients with CKD had significantly higher plasma CPP levels than healthy controls. Patients with late-stage CKD KDIGO Stage 4 and 5 were found to have the highest concentrations of plasma CPP.
**Engelbertz et al. / 2016/ Germany** [37]	Cohort	• **Total Subjects:** 301<br>• **Population:** German patients undergoing PCI<br>• **CKD:** 266 (88.4%)<br>• **Mean age (years):** CKD: 71.94±3.58; Controls: 59.5±5.02<br>• **Sex (males):** 227 (75.4%)<br>• **BMI:** All patients: 27.97±0.82<br>• **CPP Measurement Method**: EDTA blood sampling. Automated immunofluorescence assay.<br>• **CPP (pmol/L)- Mean ± SD:** Controls (*n*=35): 5.97±5.33; CKD Stage 1-2 (*n*=113): 5.53±3.23; CKD Stage 3 (*n*=117): 17.17±6.38; CKD Stage 4-5 (*n*=36): 36.70±21.77.<br>• **CPP – AUC: -**<br>• **Measurement:** Plasma	When compared to healthy controls, patients with CKD had significantly higher plasma CPP levels. Patients classified as having CKD KDIGO Stage 4-5 had the highest concentrations of plasma CPP.
**Niemczyk et al. /2018/ Poland** [40]	Prospective Cohort	• **Total Subjects:** 76<br>• **Population:** Non-Diabetic patients with PCKD<br>• **CKD:** 65 (85.5%)<br>• **Mean age (years):** CKD: 38-76yo; Controls: 38-76.<br>• **Sex (males):** - (%)<br>• **BMI:** 21-27<br>• **CPP Measurement Method**: Enzyme-linked immunoassay (ELISA) CPP (Human EIA; EK-065-32).<br>• **CPP (pmol/L)- Mean ± SD:** Controls (n=11):17.55 (11.84-32.04) ±; CKD3A (*n*=17):25.77 (20.24-34.05) ±; CKD3B (*n*=12):27.28 (19.97-44.11) ±; CKD 4: (*n*=18): 33.03 (25.19-40.41); CKD 5: (*n*=9): 41.13 (30.06-49.01); CKD 3B+HF (*n*=9) 39.69 (27.89-42.52).<br>• **CPP – AUC:**<br>• **Measurement:** Plasma	CPP is a significant marker for CKD, but its relationship to cardiac function less clear. CPP prognostic sensitivity in CKD may be improved by CPP/creatinine, CPP/cystatin C, and CPP/eGFR ratios.

**Villela-Torres et al. /2018/ Mexico** [41]	Cross Sectional Study	• **Total Subjects:** 480<br>• **Population:** Patients with T2DM and CKD<br>• **CKD:** 413 (86%)<br>• **Mean age (years):** CKD 2: 59±7 CKD 3: 62±7 CKD 4: 63±6 CKD 5: 60±9; Controls: 49±8<br>• **Sex (males):** 196(41%)<br>• **BMI:** Controls: 31.8±6.0: CKD 2: 28.6±4.0; CKD 3: 28.5±5.4; CKD 4: 26.4±4.4; CKD 5: 25.4 ±4.3.<br>• **CPP Measurement Method**: Commercial enzyme immunoassay (EK-065-32)<br>• **CPP (pmol/L)-** Mean ± SD: Controls (*n*=67):10,04 ±4.76; CKD 2 (*n*=142):12,42 ±5.37; CKD 3 (*n*=97):14.98 ±6.34; CKD 4 (*n*=31):13.46 ±5.91; CKD 5 (*n*=143):21.28±8.83.<br>• **CPP – AUC: -**<br>• **Measurement:** Plasma	High levels of plasma CPP are linked to a reduction in renal function in T2DM patients; as a result, CPP could be regarded as a biomarker of renal function.
**Alraaji et al./2020/ Iraq** [35]	Prospective Cohort	• **Total Subjects:** 84<br>• **Population:** Iraqi patients with CKD<br>• **CKD:** 44 (52.3%)<br>• **Mean age (years):** CKD: 57.87±11.42; Controls: 55.43±9.61<br>• **Sex (males):** 84 (100%)<br>• **BMI: -**<br>• **CPP Measurement Method**: ELISA<br>• **CPP (pmol/L)- Mean ± SD:** Controls (n=40):15,61±6.73; CKD (*n*=44):35.54±12,99<br>• **CPP – AUC: -**<br>• **Measurement:** Serum	In Iraqi males developing CKD, serum levels of CPP may be an indicator of renal injury.
**Nakae et al./2023/ Japan** [39]	Cross-sectional Cohort	• **Total Subjects:** 1262<br>• **Population:** Japanese people with CKD<br>• **CKD:** 201 (15.9%)<br>• **Mean age (years):** CKD Males: 64.6 ±7.4; CKD Females: 61.8 ±8.3; Controls Males57.9 ±10.4: Controls Females: 56 ±9.8<br>• **Sex (males):** 420 (33.3%)<br>• **BMI:** CKD Males:24 ±3.3; CKD Females: 22.5 ±3.3; Controls Males: 23.3 ±3; Controls Females: 21.3 ±3.2<br>• **CPP Measurement Method**: Automated KRYPTOR analyzer and time-resolved amplified cryptate emission technology assay.<br>• **CPP (pmol/L)- Mean ±** SD: Controls Males (*n*=319):6.23 ±4.45 Controls Females (*n*=742); 3.93 ±3.26; CKD Males (*n*=101):9.12 ±6.46; CKD Females (*n*=100): 5.28±4.64.<br>• **CPP – AUC: -**<br>• **Measurement:** Plasma	The CPP levels were significantly different between the two sexes. Male Patients with CKD having the highest levels followed by Females with CKD, Males without CKD and lastly females without CKD.

Chronic Kidney Disease: CKD, Copeptin: CPP, Glomerular Filtration Rate: GFR, Estimated Glomerular Filtration Rate: eGFR, Body Mass Index: BMI, Area Under Curve: AUC, Type 2 Diabetes Mellitus: T2DM, N-terminal pro-B-type natriuretic peptide: NT-pro-BNP, Growth Differentiation Factor 15: GDF-15, Dickkopf-related protein 3: DKK3, Kidney Injury Molecule-1: KIM-1, Aminopeptidase N: APN.
