# Supplementary material for: Copeptin as a Biomarker in Chronic Kidney Disease—A Systematic Review and Meta-Analysis
Source: Biomolecules. 2025 Jun 10;15(6):845. doi: 10.3390/biom15060845 (PMC12191427; doi:10.3390/biom15060845)
Supplement: Supplementary file 1 [file biomolecules-15-00845-s001.zip › Supplementary Table S2 CPP.pdf]

Supplementary Table 2. The Newcastle-Ottawa Scale (NOS) for assessing the quality of cross-sectional studies

**Study**	**Selection** (Maximum 5 stars)				**Comparability** (Maximum 2 stars)	**Outcome** (Maximum 3 stars)		**Score** (Total maximum 10 stars)
	**Sample representativeness**	**Sample size**	**Non-Respondents**	**Ascertainment of the exposure (risk factor)**	**Comparability**	**Assessment of the outcome**	**Statistical test**	
Li et al 2013 [38]	*	*	-	**	-	**	*	7
Bjurman et al 2015 [36]	*	*	-	**	-	*	*	6
Engelbertz et al 2016 [37]	*	*	*	**	-	**	*	8
Niemczyk et al 2018 [40]	-	-	-	**	-	**	*	5
Viella-Torres et al 2018 [41]	-	-	-	**	-	**	*	5
Alaaraji et al 2020 [35]	-	-	-	**	-	**	*	5
Nakae et al 2023 [39]	*	*	-	**	-	**	*	7
